# Simulating the impact of piers on hydrodynamics and pollutant transport: A case study in the Middle Yangtze River

**DOI:** 10.1371/journal.pone.0260527

**Published:** 2021-12-01

**Authors:** Haibin Xiong, Li Chen, Zhaohua Sun, Zhiqing Li, Kun Zhou, Zhenghao Chen

**Affiliations:** State Key Laboratory of Water Resources and Hydropower Engineering Science, Wuhan University, Wuhan, China; University of Parma: Universita degli Studi di Parma, ITALY

## Abstract

It is known that channel engineering, including the construction of piers, will change the river hydrodynamic characteristics, which is a significant factor affecting the transport process of pollutants. With this regard, this study uses the well-validated and tested hydrodynamic module and transport module of MIKE 21 to simulate the hydrodynamics and water quality under various pier densities in the Wuhan reach. Hydrodynamic changes around the piers show spatial differences, which are similar under different discharges. The range and amplitude of hydrodynamic spatial variations increase with the increase in pier density. However, there is a critical value of 1.25 to 2.5 units/km. When the pier density is less than this critical value, this type of cumulative effect is the most significant. Additionally, greater changes can be found in chemical oxygen demand concentrations, which also show spatial and temporal variations. The area with high chemical oxygen demand concentration upstream and downstream from the engineering area exhibits the distribution characteristics of “decrease in the downstream area and increase in the upstream area” and “increase in downstream the area and decrease in the upstream area” respectively. In the reach section of the engineering area, the area with high chemical oxygen demand concentration increases in the front area near the piers and decreases near the shoreline. Furthermore, the concentration shows attenuation actions with a longer residence time owing to the buffering effect of pier groups. These results have significant implications on shoreline planning and utilization. Moreover, they provide scientific guidelines for water management.

## Introduction

Owing to increasing anthropogenic pressures (e.g., urban expansion, recreational development, channel engineering, flood defense, etc.), shoreline changes can be observed worldwide [1]. For example, ports (port engineering) are one of the main drivers of change in the shoreline area of the middle and lower reaches of the Yangtze River, which has long been known as the Golden Waterway and served as an important link in the construction of the Yangtze River Economic Belt [2]. Furthermore, the number of sudden water pollution accidents has increased rapidly with industrial development [3]. On September 24, 2008, a drainage pipe of a chemical plant in Xinyang broke and released a large amount of wastewater directly into the Yangtze River, polluting 11 km of the river near the leakage location and affecting the farmland irrigation of five surrounding villages. On November 13, 2005, an aniline production factory of Jilin Petrochemical Corporation released 100 tons of benzene substances into the Songhua River. This accident cut off the main water supply of Harbin City, which is located downstream from the accident site, for several days [4]. Throughout the large-scale utilization of the shoreline. However, the impacts of port construction on the movement of pollutants due to water pollution incidents in rivers have been neglected in the past.

Research on port engineering, especially piers, focuses on hydrodynamic characteristics, such as backwater effects and flow fields near hydraulic structures [5, 6]. These studies provide a detailed understanding of the effects of piers on flow of water. Although few studies have examined the cumulative effects on hydrodynamics caused by pier groups, they mainly concentrated on flood issues. In addition, Investigations of water quality research, including sudden water pollution, tend to focus on the spatiotemporal concentrations of pollutants by analyzing water quality monitoring data or using numerical models [7, 8]. These results are helpful for identifying water quality changes and assessing pollution problems. Furthermore, some studies have focused on the effects of hydrodynamic changes on water quality. For example, Long et al. [9] conducted numerical simulations to study how the movement and diffusion of pollutants changed after dams were built on the Xiangjiang River. Jia et al. [10] analyzed the influence of coastal shoreline changes on the hydrodynamics and water quality of Bohai Bay using the hydrodynamic module (HD) and Ecolab module of MIKE21. In contrast to coastal areas, hydrodynamic factors, such as velocity and flow direction, are severely affected by the river boundary. Moreover, the main drivers of pollutant transport downstream are currents. However, to the best of our knowledge, no research has been conducted to examine the role of piers in the transport of solute pollutants in inland rivers, especially those resulting from sudden water pollution accidents.

To fill this gap, we chose the Wuhan reach as a representative of urban river sections of the Yangtze River for two reasons. On one hand, the Wuhan reach has rich shoreline resources with a large number of piers. On the other hand, it has high flood control pressure and water quality requirements. Although Zhang et al. [11] studied the hydrodynamic effects of piers in this reach, the simulation of sudden water pollution incidents were performed in previous investigations [12]. To date, the aforementioned studies did not provide any connection between piers and sudden water pollution, but rather provided insight into the effect of pier structure on pollutant transport. Although Xiong et al. [13] studied the impact of piers on oil spill transport in this channel, knowledge gaps still exist regarding dissolved pollutants, which are completely different from leaked oil. In particular, issues, such as the difference in hydrodynamic changes during flood and dry periods caused by different pier densities and the relationship and difference in the changes between hydrodynamic conditions and pollutant transport due to the construction of piers, have not yet been discussed systematically.

The objective of the this study is to assess the variability in hydrodynamics and dissolved pollutant transport of the urban river with several ports distributed along the shoreline based on the analysis of the variations in water level, flow velocity, and characteristics of pollutant transport (peak concentration, arrival time, and residence time) in Wuhan. A detailed understanding of the influence of piers on the flow regime and pollutants is not only helpful in developing response strategies for water resource protection when flood hazards or sudden water pollution accidents occur near the port area, but also offer advice for shoreline planning and utilization. For instance, allowing the construction of dense piers in areas that have less effect on flooding and water quality instead of areas having a significant effect on the aforementioned factors.

## Materials and methods

### Study area

**Fig 1:**
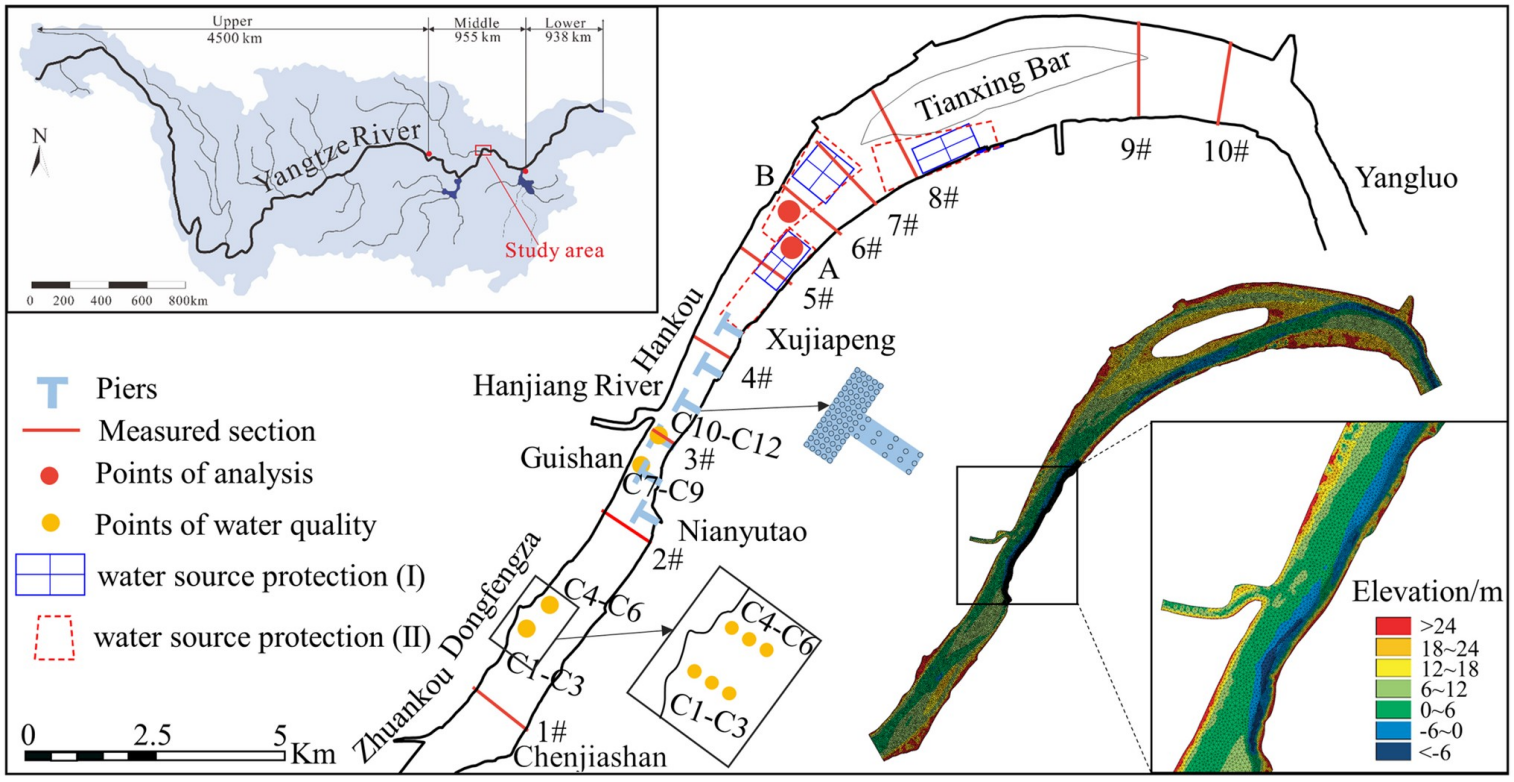
Map of the study area. Locations of sampling sites, hydrological cross-sections, and model mesh.

The Yangtze River is divided into the upper, middle, and lower reaches, which are 4505 km, 955 km, and 938 km in length, respectively, based on different geographical and hydrological characteristics [14]. The Wuhan reach is located in the Middle Yangtze River, which is 70.3 km in length. The middle and lower sections of this reach were chosen as the study area because there are many piers on both sides of the river. The study reach, i.e., the Zhuankou to Yangluo reach (ZYR), extends from Zhuankou to Yangluo and has a total length of 50.6 km, as shown in Fig 1. The upper section of ZYR, which extends from Zhuankou to Guishan (15.2 km), has a relatively straight channel pattern. The lower ZYR is classified as a slightly curved and branched channel extending from Guishan to Yangluo. The width of the river at Zhuankou is approximately 1450 m, whereas that at Yangluo is approximately 1400 m. The narrowest and widest part of the river are near Guishan and head of Tianxing bar with a total width of 1100 m and 4000 m, respectively. The Hanjiang River, a considerable tributary, which is located on the left bank of this stretch, converges into the main river at Hankou [15].

ZYR flows through Wuhan City, which is the megacity of central China with a population of tens of millions, and is the main water source of this city. The water quality standards of the first (I) and second (II) water source protection areas in ZYR should not be lower than the Chinese water surface water levels II and III, respectively. The Nianyutao–Xujiapeng shoreline (NXS) with a length of 9.6 km was selected as a typical shoreline because the width of this shoreline is narrow, which makes it easy for the flow to interfere after pier construction. Based on numerical experiments, we explored the effect of arranging piers with different densities in the NXS on the hydrodynamics and dissolved pollutant transport within an idealized ZYR. Because the transportation laws of various types of pollutants are similar, the commonly used chemical oxygen demand (COD) was selected as the representative pollutant in this study [16].

### Numerical model

#### Governing equations

MIKE21 FM, created by the Danish Hydraulics Institute, has been widely applied in scientific and environmental consulting communities [17, 18]. We performed simulations using the MIKE21 FM-coupled HD and transport module (TR). The HD module is based on the numerical solution of the two-dimensional shallow water equations, which are composed of the continuity equation and the horizontal momentum equations. The TR module, based on the advection-dispersion equations, calculates the resulting transport of materials based on the flow conditions provided by the HD module. The governing equations in Cartesian co-ordinate are given by

$$\frac{\partial h}{\partial t}+\frac{\partial h\bar{u}}{\partial x}+\frac{\partial h\bar{v}}{\partial y}=hS$$ (1)

$$\begin{array}{l} \frac{\partial h\bar{u}}{\partial t}+\frac{\partial h{\bar{u}}^{2}}{\partial x}+\frac{\partial h\bar{vu}}{\partial y}=f\bar{v}h-gh\frac{\partial \eta }{\partial x}-\frac{h}{\rho_{0}}\frac{\partial p_{a}}{\partial x}-\frac{gh^{2}}{2\rho_{0}}\frac{\partial \rho }{\partial x}+\\ \frac{\tau_{sx}}{\rho_{0}}-\frac{\tau_{bx}}{\rho_{0}}-\frac{1}{\rho_{0}}\left(\frac{\partial s_{xx}}{\partial x}+\frac{\partial s_{xy}}{\partial y}\right)+\frac{\partial }{\partial x}\left(hT_{xx}\right)+\frac{\partial }{\partial y}\left(hT_{xy}\right)+hu_{s}S \end{array}$$ (2)

$$\begin{array}{l} \frac{\partial h\bar{v}}{\partial t}+\frac{\partial h{\bar{v}}^{2}}{\partial y}+\frac{\partial h\bar{uv}}{\partial x}=-f\bar{u}h-gh\frac{\partial \eta }{\partial y}-\frac{h}{\rho_{0}}\frac{\partial p_{a}}{\partial y}-\frac{gh^{2}}{2\rho_{0}}\frac{\partial \rho }{\partial y}+\\ \frac{\tau_{sy}}{\rho_{0}}-\frac{\tau_{by}}{\rho_{0}}-\frac{1}{\rho_{0}}\left(\frac{\partial s_{yx}}{\partial x}+\frac{\partial s_{yy}}{\partial y}\right)+\frac{\partial }{\partial x}\left(hT_{xy}\right)+\frac{\partial }{\partial y}\left(hT_{yy}\right)+hv_{s}S \end{array}$$ (3)

$$\frac{\partial h\bar{\text{C}}}{\partial t}+\frac{\partial h\bar{u}\bar{C}}{\partial x}+\frac{\partial h\bar{v}\bar{C}}{\partial y}=\frac{\partial }{\partial x}\left(hE_{x}\frac{\partial \bar{C}}{\partial x}\right)+\frac{\partial }{\partial y}\left(hE_{y}\frac{\partial \bar{C}}{\partial y}\right)-hk\bar{C}+hC_{s}S$$ (4)

Where *h* is the total water depth; $\bar{u}$ and $\bar{v}$ are the depth-average velocity at *x* and *y* direction, respectively; *S* is the magnitude of the discharge due to point sources; *f* is the Coriolis parameter; *η* is the surface elevation; *τ*_*sx*_ and *τ*_*sy*_ are the surface wind stresses; *τ*_*bx*_ and *τ*_*by*_ are the bottom stresses; *s*_*ij*_ are the radiation stress tensor; *T*_*ij*_ are the lateral stress; $\bar{\text{C}}$ is the concentration of the pollutant; *k* is the linear decay rate; *E*_*x*_ and *E*_*y*_ are the sum of diffusion coefficient and dispersion coefficient in the x and y direction, respectively; *C*_*s*_ is the concentration of the pollutant at the source.

The discretization in solution domain is performed using a cell-centered finite volume method. A second order Runge Kutta method was used for time integration. More detailed descriptions of numerical schemes can be obtained from the official scientific documentation of MIKE 21 [19].

#### Boundary conditions and parameters

The model domain covered the entire ZYR and shoreline regions. The shoreline boundary and topographic data were extracted from a 1:10,000 scale map of 2011. The computational mesh was developed using the mesh generator provided by the MIKE21 software. The final mesh, which adopted an unstructured mesh, was composed of 46,563 elements and 23,254 nodes with local refinement in the engineering area along the NXS. The spatial resolution ranged from 50~20 m in the main channel with the minimum grid size of 2 m in the refined area. The model mesh and bottom elevation of ZYR are shown in Fig 1.

The discharge and COD concentrations of the Zhuankou station and Hanjiang River were specified as the inflowing boundaries. The water levels of Yangluo station were specified as downstream boundaries with a concentration of zero gradient. The initial water surface levels were set to be slightly higher than those at the downstream boundary, and the initial velocities were set to be zero over the domain. The initial COD concentrations were determined based on the observed data in ZYR.

The Courant-Friedrichs-Lewy number was set as 0.8, and the depth for drying, flooding and wetting were set as 0.005 m, 0.5 m and 0.1 m, respectively. The wind was not considered owing to its weakness. The eddy viscosity type has been chosen to Smagorinsky formulation and the coefficient was set to 0.28, in accordance with previous studies [16, 20]. A computational time step of 30 s was chosen in this study.

#### Model calibration and verification

The root mean square error (RMSE) and Nash-Sutcliffe efficiency coefficient (NSE) were used to assess the accuracy of the model [18, 21]. 5 observed hydrological data set (including water level and velocity) at the fixed cross-sections in 2009–2011 were collected for HD module, 2 were for calibrating the model and 3 for verification. As for the TR module, the monitoring data of water quality (COD concentration) in 2011 and 2016 downstream of different sewage outlet were used for calibration and verification, respectively. The location monitoring sections and points are shown in Fig 1. Specifically, The Manning’s n in the HD module were calibrated using the measured data on August 2011 and March 2009. On the basis of running HD module, the parameters of Ex, Ey and k in TR module were adjusted using filed water quality data on November 2011. The boundary conditions in each case are shown in Tables 1 and 2. We adjusted this parameter until the RMSE values reached a minimum, the calibration results of water level, velocity and COD are given in S1–S5 Figs.

**Table 1:** Boundary conditions of HD module in calibration and verification stages.

Stage	Discharges at Zhuankou (m^3^/s)	Discharges at Hanjiang (m^3^/s)	Water level at Yangluo (m)	field data (year.month)
**calibration**	30100	1700	18.6	2011.08
	12000	700	13.2	2009.03
**verification**	12971	656	13.7	2011.11
	25150	1450	18.3	2011.07
	37950	2050	22.2	2010.08

**Table 2:** Boundary conditions of TR module in calibration and verification stages.

Stage	Sewage outlet	Sewage flow (m^3^/s)	Sewage COD (mg/L)	COD at Zhunakou and Hanjiang (mg/L)	Initial COD (mg/L)	field data (year.month)
**calibration**	Chenjiashan	3.73	28.5	3.2	3.2	2011.11
**verification**	Dongfengza	9.76	63.5	8.1	8.1	2016.10

After calibration, it was found that the values of Manning’s n across the study reach range from 50 to 65 m^1/3^/s with the upstream incoming flow from dry to flood discharge. In addition, the values of the coefficient in the TR model were determined as Ex = Ey = 0.5~0.6 m^2^/s and k = 2.89×10^−6^ 1/s, which are close to the measured values of Changjiang Water Resources Protection Institute in the same reach. [22].

**Fig 2:**
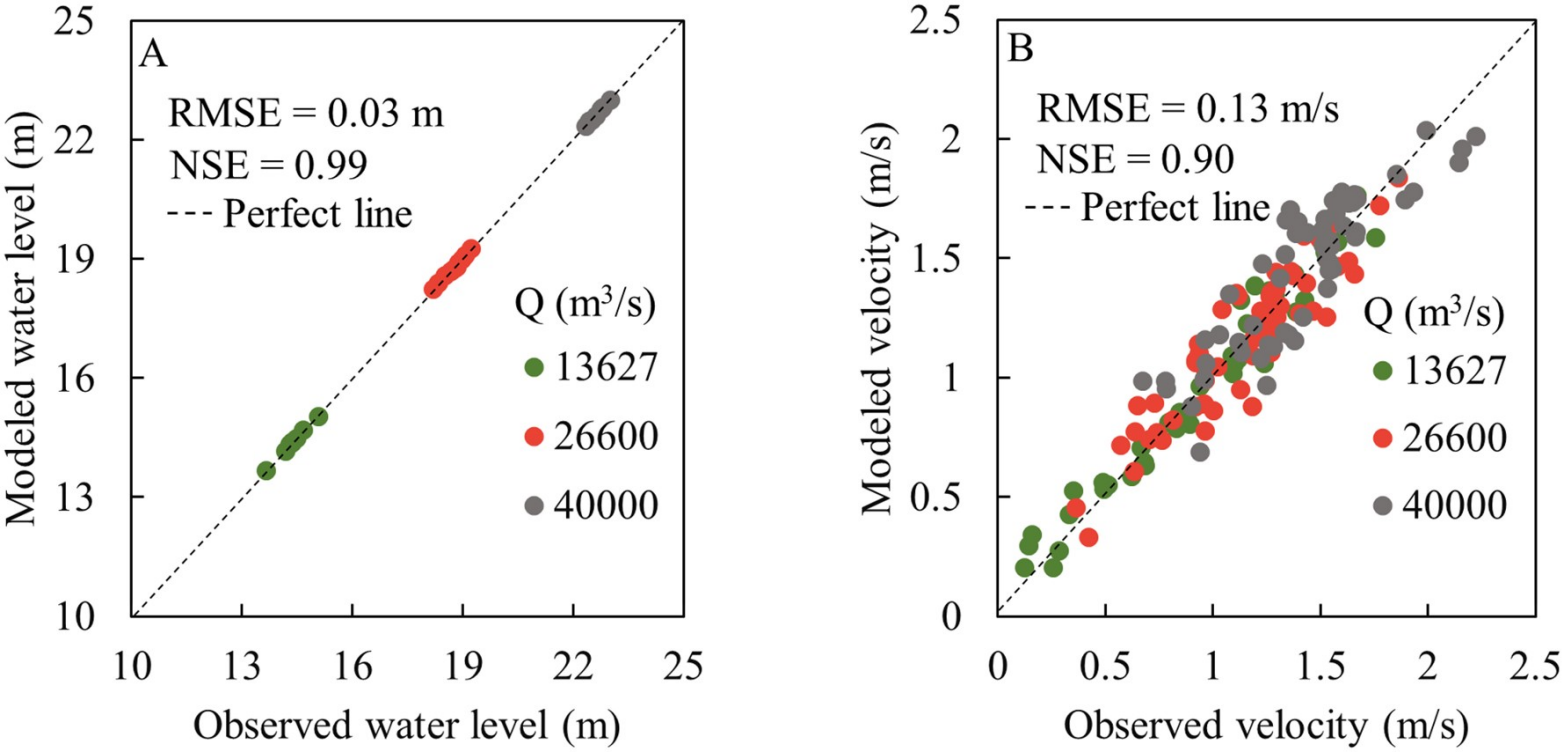
Model verification of (A) water level and (B) depth averaged velocity under various discharges.

**Fig 3:**
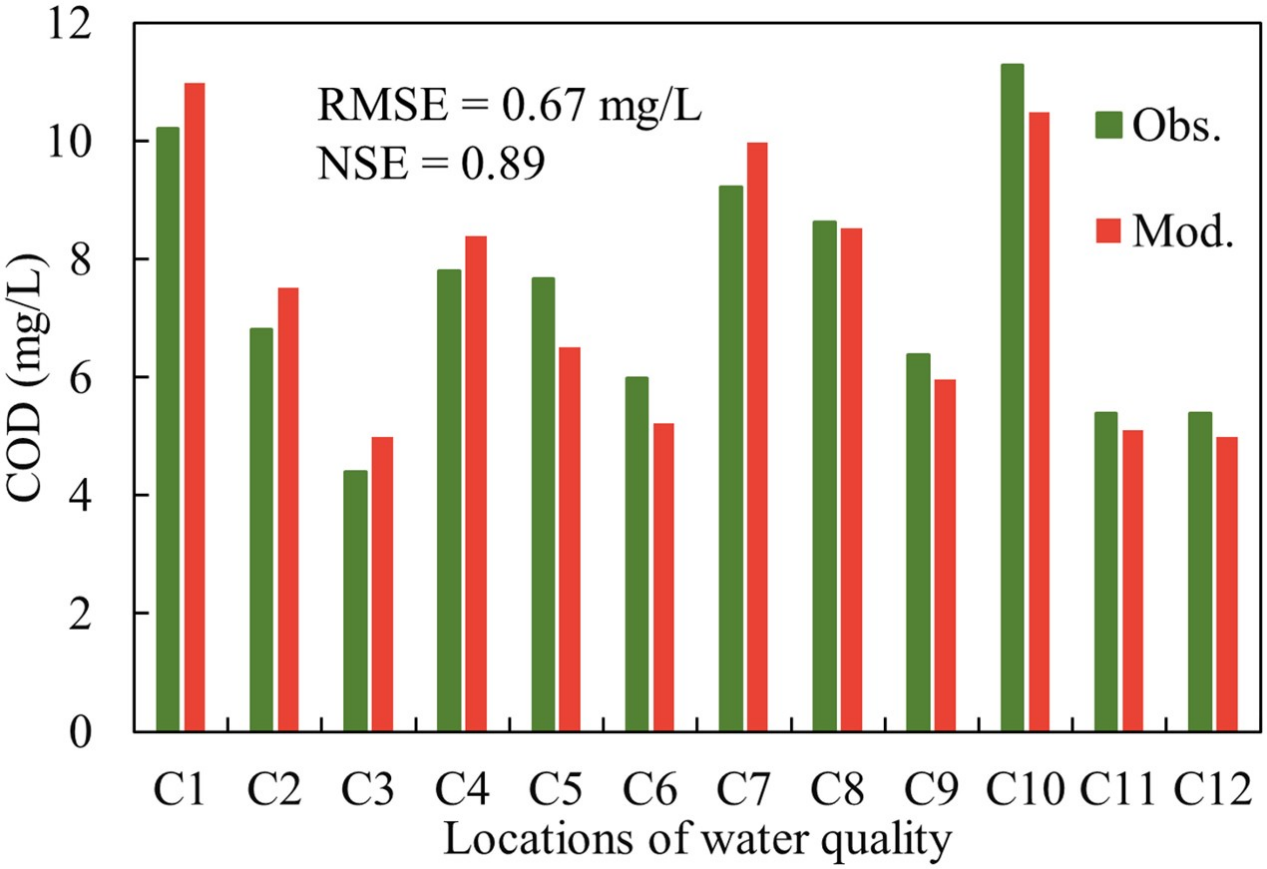
Comparison of modeled and observed COD concentrations.

Subsequently, the verification of the model has been done with field data to assess the efficiency of the model. Referring to the relevant figure in Zhang et al. [23] and Liu et al. [24]. Fig 2 presents the correspondence between the model results and measurement of the hydrodynamics. The overall RMSE of the water level was 0.03 m, and the RMSE of the velocity was 0.13 m/s. Furthermore, the overall NSE values of the water level and velocity were 0.99 and 0.90, respectively, indicating that the modeled hydrodynamics performed almost perfectly. As shown in Fig 3, the modeled COD concentrations were compared with the observations recorded at the monitoring points. According to the performance statistics, the RMSE and NSE were 0.67 mg/L and 0.89, respectively, suggesting a satisfactory agreement. The verification results indicated that the HD and TR models reflected the hydrodynamic and pollutant transport of ZYR accurately.

#### Generalization of piers

The pier size in the model refers to the relevant parameters of the existing piers in ZYR. The size of the platform was uniformly set to be 100 m × 20 m, whereas the approach bridge was set to be 150 m × 15 m. Under the platform and approach bridge, there were 13 × 5 piles and 13 × 1 piles, respectively, having a diameter of 1.5 m. The piers were uniformly distributed along the NXS shoreline in a T-shape (Fig 1), and a single pier was generalized using the pier structure in the HD module. The effect of piers on the flow was modeled by calculating the additional drag force of each individual pier, which is widely used in practical engineering [25, 26]. The following equation shows the drag force representing piers:

$$\text{F}=0.5\rho_{\text{w}}\gamma {\text{C}}_{\text{D}}{\text{A}}_{\text{C}}{\text{V}}^{2},$$ (5)

Where ρ_w_ is the water density, γ is the streamline coefficient with a constant value of 1.03 [26], C_D_ is the drag coefficient, A_C_ is the effective water blocking area of the pier, and V is the flow velocity. The values of C_D_ and A_c_ are related to the shape and direction of the piers, submergence degree and relative water depth, etc. and has been stored in the database. During numerical simulation, only relevant data such as the position and size of the piers need to be provided.

#### Simulation scheme

Wang [27] found that when the density of piers was greater than 1.33 units/km, the influence of the pier on hydrodynamics will interfere strongly. Based on this understanding, densities of 0.31, 0.62, 1.25, 2.5, and 5 units/km were considered in this study (i.e., 3, 6, 12, 24, and 48 piers were evenly arranged along the NXS shoreline for a length of 9.6 km). Together with the natural condition (no piers), it is composed of six densities, which have a degree of generalization, but to some extent reflect the current sparse pier distribution and the possible construction of dense pier groups in the future. Three typical discharges of 39,600 m^3^/s, 22,600 m^3^/s, and 12,700 m^3^/s were selected from flood to dry flows to study the impact of various discharges on hydrodynamics (Table 3). As the dry season is the weakest period of river self-purification, the simulation of pollutant transport focuses on a flow level of 12,700 m^3^/s. The average COD concentration of 12 months was considered as the initial concentration with a value of 10.1 mg/L based on the observed data of 2017 in the study reach. We assumed an accident of COD leakage in the upper reaches of ZYR when the model was stable. The total amount of COD pollutants was set to be 2743.2 t for a period of 1 h with reference to an accident prototype, and the COD concentration was set as 63.5mg/L during the accident.

**Table 3:** Simulation scheme for HD and TR module.

Module	Discharge at Zhuankou (m^3^/s)	Discharge at Hanjiang (m^3^/s)	Water level at Yangluo (m)	Pier densities (units/km)
**HD**	12000	700	13.2	0, 0.31, 0.62, 1.25, 2.5, 5
	21380	1220	17.0	0, 0.31, 0.62, 1.25, 2.5, 5
	37840	1760	22.1	0, 0.31, 0.62, 1.25, 2.5, 5
**TR**	12000	700	13.2	0, 0.31, 0.62, 1.25, 2.5, 5

The statistics and analysis of the results follow the overall criterion for partial thinking, i.e., analyze the change characteristics of the entire reach first and then focus on exploring the detailed hydrodynamic and pollutant transport changes in the waters near the engineering area, especially the change in water quality in the water source protection area. We selected two typical locations on the left and right water-source protection areas, i.e., locations A and B, as shown in Fig 1. Velocity and water level are considered as indicators for the change in hydrodynamics. The changes in pollutant transport are based on the spatial distribution of COD concentration, peak concentration, arrival time of the peak concentration, and residence time. To compare the hydrodynamics and pollutant transport changes under different pier densities, the relative changes in flow velocity, water level (water depth), and COD concentration were used as indicators.

## Results

### Changes in hydrodynamics

**Fig 4:**
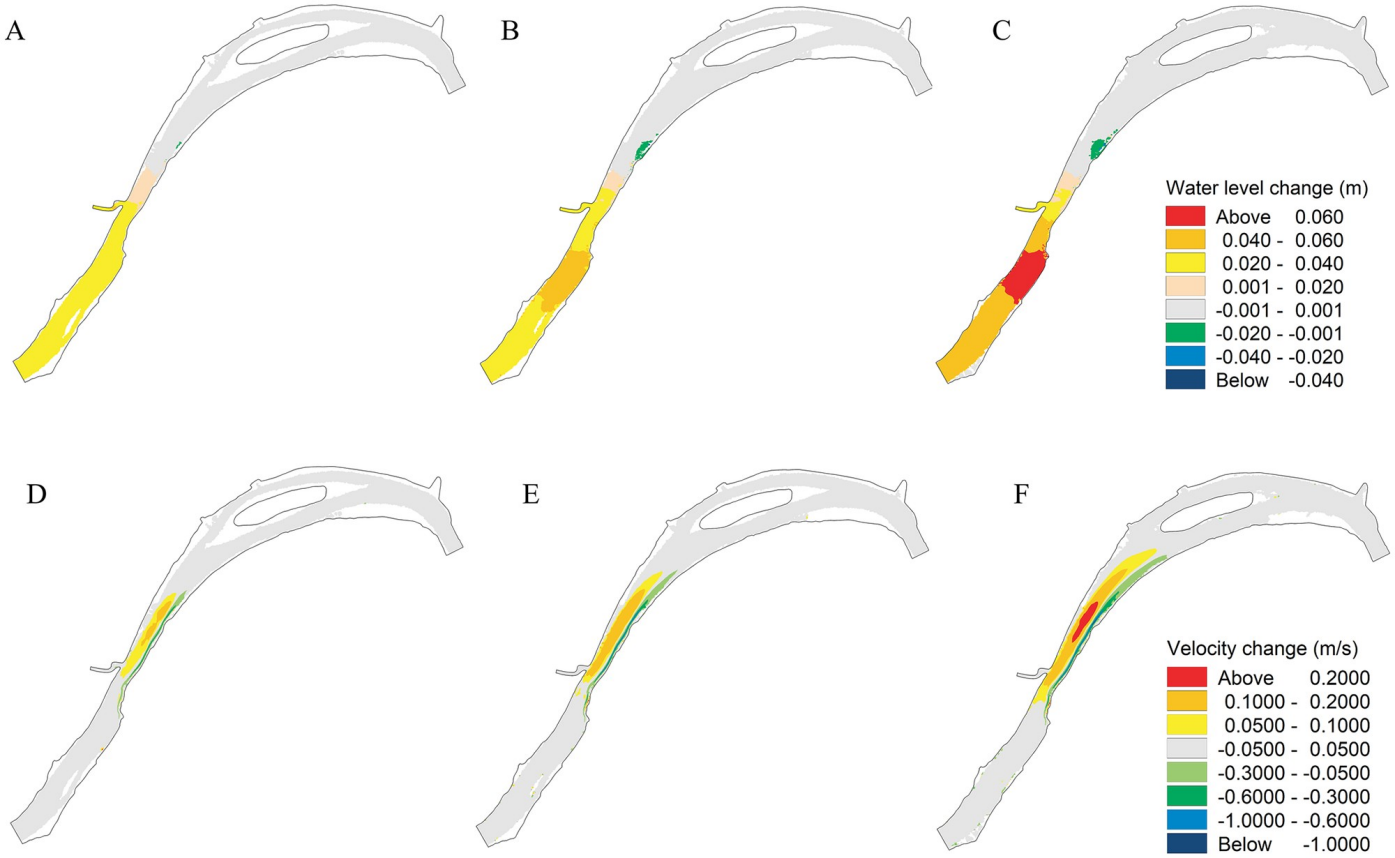
Change in hydrodynamics with and without piers under different discharges. (A), (B), and (C) represent the water level change under the discharge of 12,700 m^3^/s, 22,600 m^3^/s, and 39,600 m^3^/s, respectively; (D), (E), and (F) represent the velocity change the discharge of 12,700 m^3^/s, 22,600 m^3^/s, and 39,600 m^3^/s, respectively.

According to the analysis, the flow conditions of different pier densities were similar. Consider the maximum density of 5 units/km as an example. The differences in the water level and velocity spatial distributions with and without piers under various discharges are presented in Fig 4. Rise in water levels were observed upstream of the engineering area. The influence was more obvious under flood discharge with a maximum increase of 0.07 m. However, the water level at the tail edge of the engineering area decreased slightly. Owing to the backwater effect of pier groups, a narrow and long flow velocity-decrease belt was formed in the engineering area, like the water level, the spatial range and amplitude of the velocity change increased with the increase in flow rate. The maximum reduction of the velocity in the flood discharge was 1.12 m/s. In contrast, the front water area of the engineering area formed a large-scale flow velocity-increase area with a maximum increase of 0.24 m/s.

### Changes in pollutant transport

#### Change in pollutant transport in the study reach

**Fig 5:**
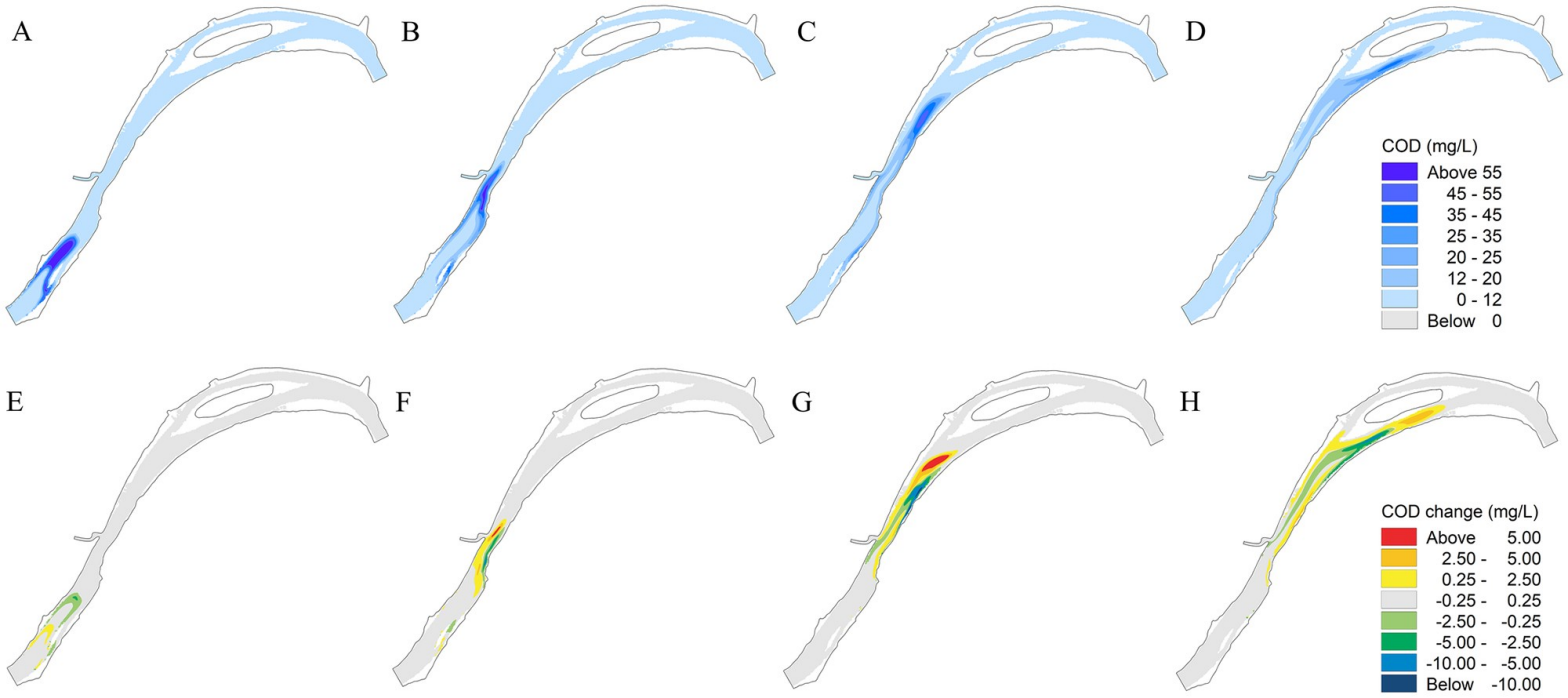
COD concentration distribution without piers and change in COD concentration with and without piers. (A), (B), (C), and (D) represent the time of 2.5 h, 4.5 h, 6.5 h, and 8.5 h; (E), (F), (G), and (H) represent the time of 2.5 h, 4.5 h, 6.5 h, and 8.5 h.

Fig 5A–5D show the COD spatial distribution at 2.5 h, 4.5 h, 6.5 h, and 8.5 h when there are no piers. It can be observed that the transportation of high-concentration pollutants under natural conditions is the same as that of the mainstream of the river, and the concentration decreases gradually as COD transports downstream. Due to the slow velocity in near-shore waters, long and narrow high-concentration areas are formed on both sides of the river. Fig 5E–5H show the difference in COD concentration with and without piers (like the hydrodynamics, taking the density of 5 units/km as an example). There are obvious areas of increase and decrease in COD concentration after the construction of piers, and the location and scale gradually changes over time. Before the area with high COD concentration moved to the engineering area (2.5 h), the distribution of COD concentration decreased in the downstream area and increased in the upstream area. The variation in COD concentration ranged from −5.9 to 3.0 mg/L. When the area with high COD concentration moved to the reach of the engineering area (4.5 h), the distribution of COD concentration increased in the front area near the piers and decreased near the shoreline, where the flow velocity varied with the change in COD concentration ranging from −8.7 to 7.5 mg/L. When the area with high COD concentration moved outside the reach of the engineering area (6.5 h), the distribution of the COD concentration increased in the downstream area and decreased in the upstream area with the change ranging from −14.3 to 11.1 mg/L. This is because the transportation of the pollutant accelerates with the increase in flow velocity in the mainstream area, whereas the decrease in flow velocity near the shoreline of the engineering area reduces the migration rate of the pollutant, resulting in the increase in COD concentration at 8.5 h.

#### Changes in pollutant transport in a typical section

**Fig 6:**
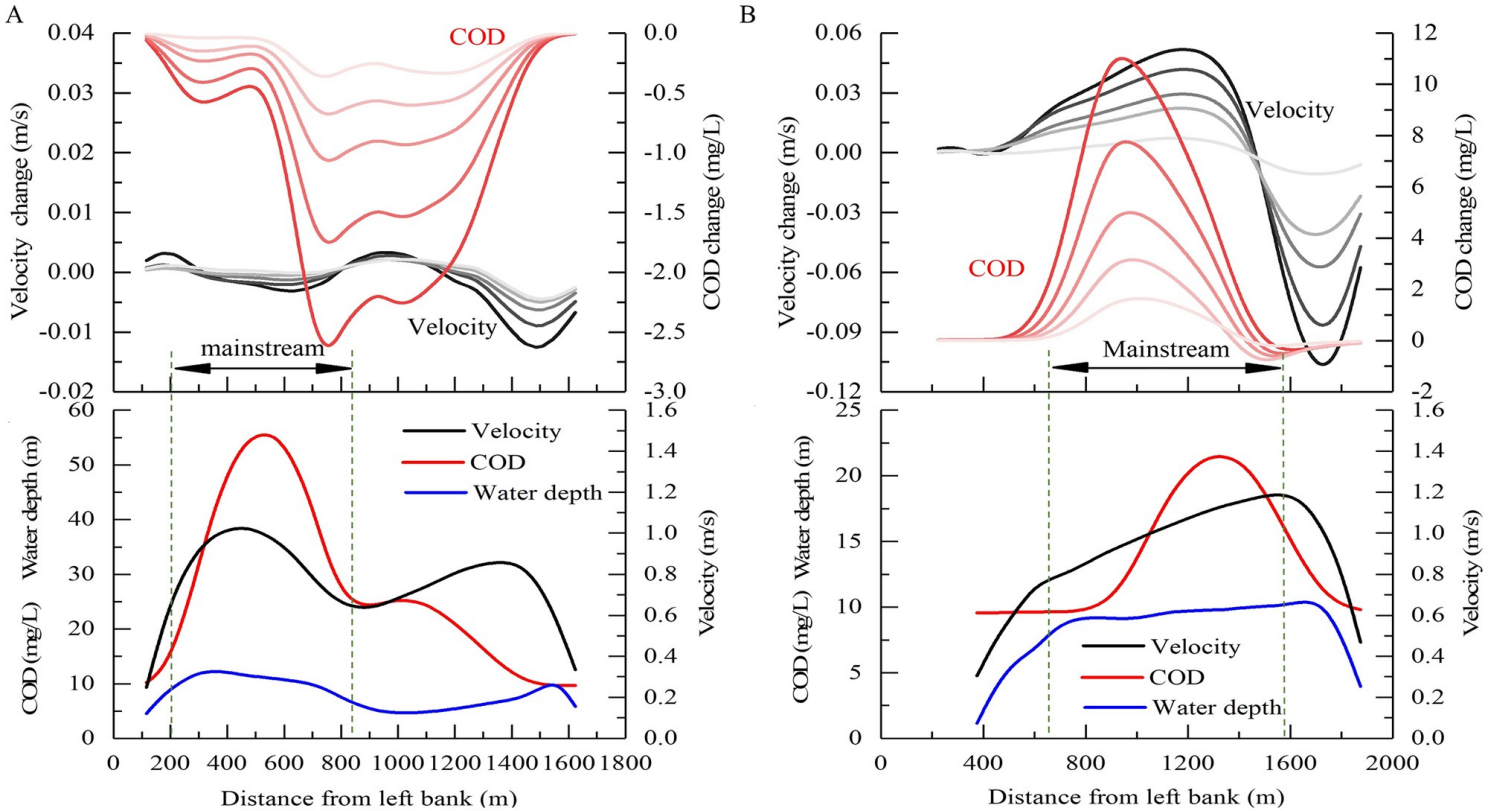
Change in lateral distribution of COD concentration and flow velocity with and without piers. (A) 2# cross-section at the time of 3.275 h and (B) 5# cross-section at the time of 5.95 h. The solid line indicates the pier densities of 0.31, 0.62, 1.25, 2.5, and 5 units/km as the color deepens, respectively.

The cross-sections of 2# and 5# were located near the upstream and downstream of the engineering area, respectively (Fig 1). The reach between the two cross-sections was considered as the typical river section. For the two phenomena of decrease and increase in area with high COD concentration, as shown in Fig 5, the moments with the most obvious changes in COD concentration were selected. Fig 6 shows the flow velocity and change in average COD concentration along the cross-section at the time of 3.275 h and 5.95 h. It can be observed that, similar to the changes in hydrodynamic conditions, the variation in COD concentration will increase with an increase in the pier density. However, the position where the COD concentration changes the most and the position where the flow velocity changes the most do not coincide. The former is mainly concentrated in areas with great depth and high velocity in the middle of the river, whereas the latter is located near the shoreline of the engineering area.

**Fig 7:**
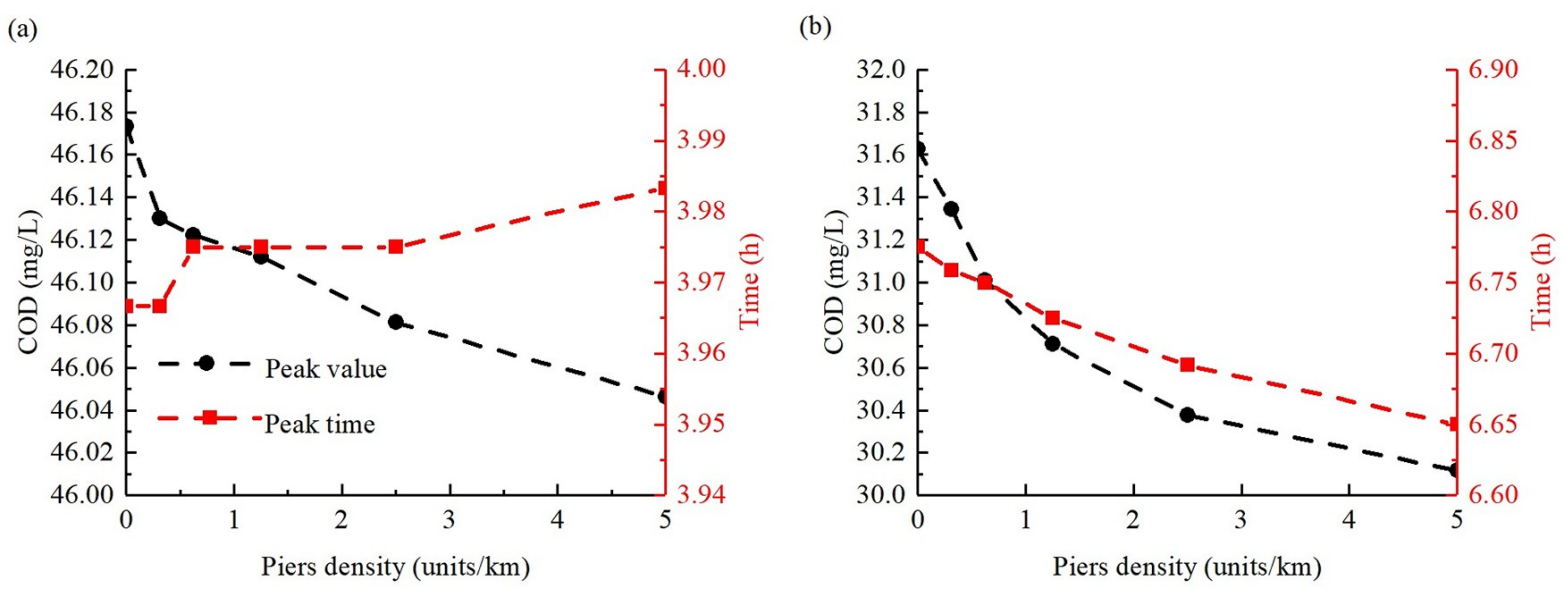
Change in average COD concentration peak value and arrival time at different cross-sections. Change in average COD concentration peak value and arrival time at (A) 2# cross-section and (B) 5# cross-section under different pier densities.

Fig 7 shows the COD peak concentration and the time when the peak reached the cross-sections of 2# and 5# (Fig 1). It can be observed that the peak concentration of COD in cross-section 2# decreased gradually with the increase in pier density, and the maximum decrease was 0.13 mg/L. Furthermore, the peak arrival time was delayed with a maximum value of 0.02 h. The peak concentration of COD in cross-section 5# also decreased with an increase in the pier density, and the maximum decrease was 1.5 mg/L. The difference is that the peak arrival time will be earlier with a maximum value of 0.12 h in cross-section 5#. The reason for the aforementioned phenomenon is that the flow velocity of the upstream cross-section 2# decreases owing to the impact of backwater, whereas the flow area of the section where the pier group is located is squeezed, resulting in an increase in the flow rate. This delays the time required for high-concentration pollutants to move to the engineering reach, and when the high-concentration pollutants arrive at the engineering reach, they move downward and accelerate. This effect becomes more obvious as the pier density increases. The decrease in the peak value is due to the existence of pier groups, which decreases the nearshore velocity significantly (Fig 6), buffers the change in the average concentration of the cross-section scale, and thereby reduces the fluctuation process of the pollutant concentration of the cross-section. This is similar to the homogenized discharge process due to reservoir operation [28].

**Fig 8:**
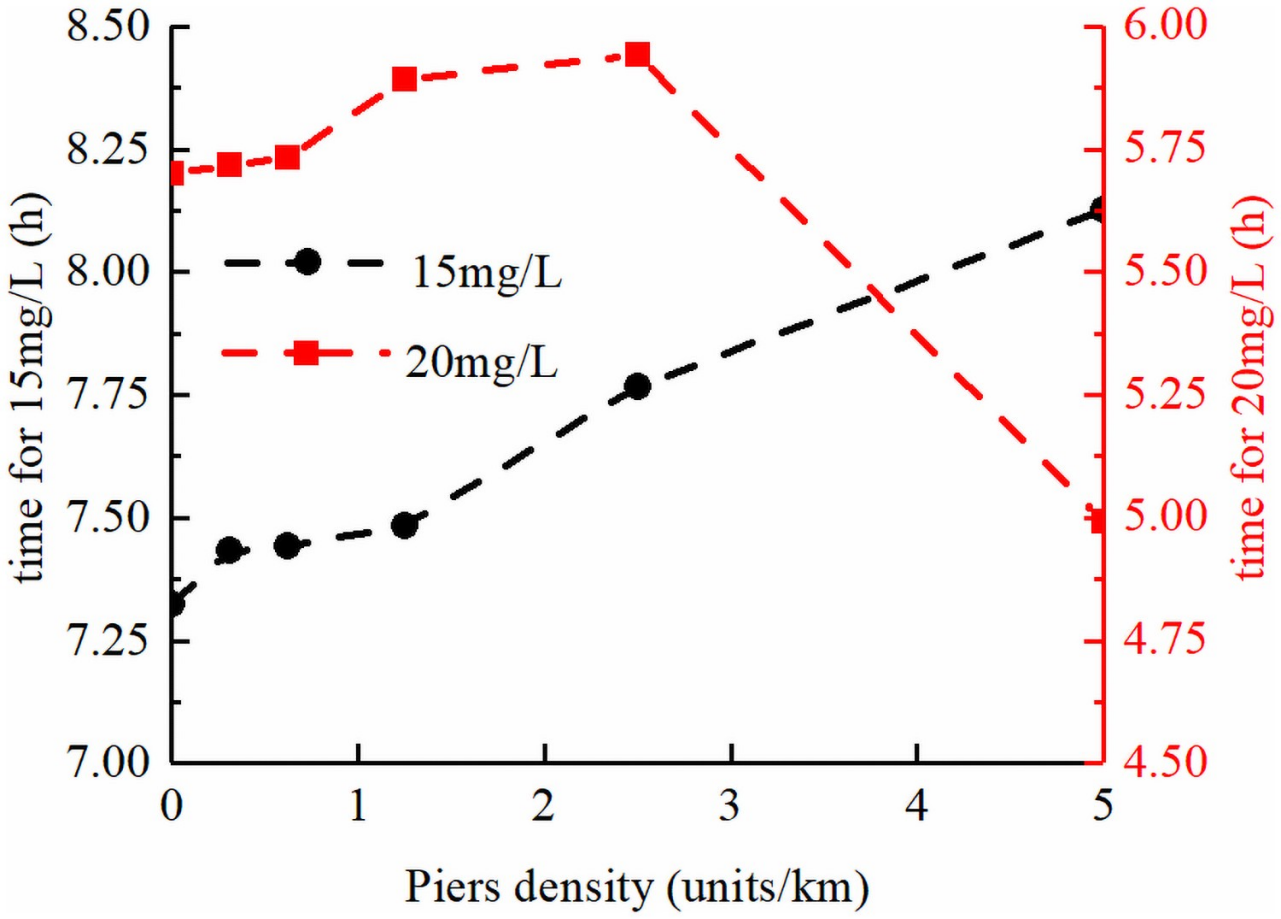
Variation in residence time of COD in the representative reach section under different pier densities.

Fig 8 shows the residence time of COD concentrations exceeding level II and III water quality (15 mg/L and 20 mg/L, respectively) in the reach section between cross-section 2# and 5#. The calculation of time starts when any grid concentration exceeds the critical value, and ends when all grid concentrations are lower than the critical value. As shown in Fig 8, for level II water quality, compared with the case of no piers, the residence time of COD concentration in the reach section increased gradually with the increase in pier density. The maximum increase time was 0.8 h. However, for COD level III water quality, it was found that after the pier density increased to a certain level, the residence time decreased. The reason for this phenomenon is similar to that shown in Fig 7, i.e., the near-shore flow velocity reduction area has a buffering effect on the overall pollutant transport process, thereby widening (lowering) the peak and lengthening the process. The law observed in Fig 8 also shows that this phenomenon of peak flattening exists regardless of the average of the cross-section scale or grid scale in the plane.

#### Changes in pollutant transport in typical locations

**Fig 9:**
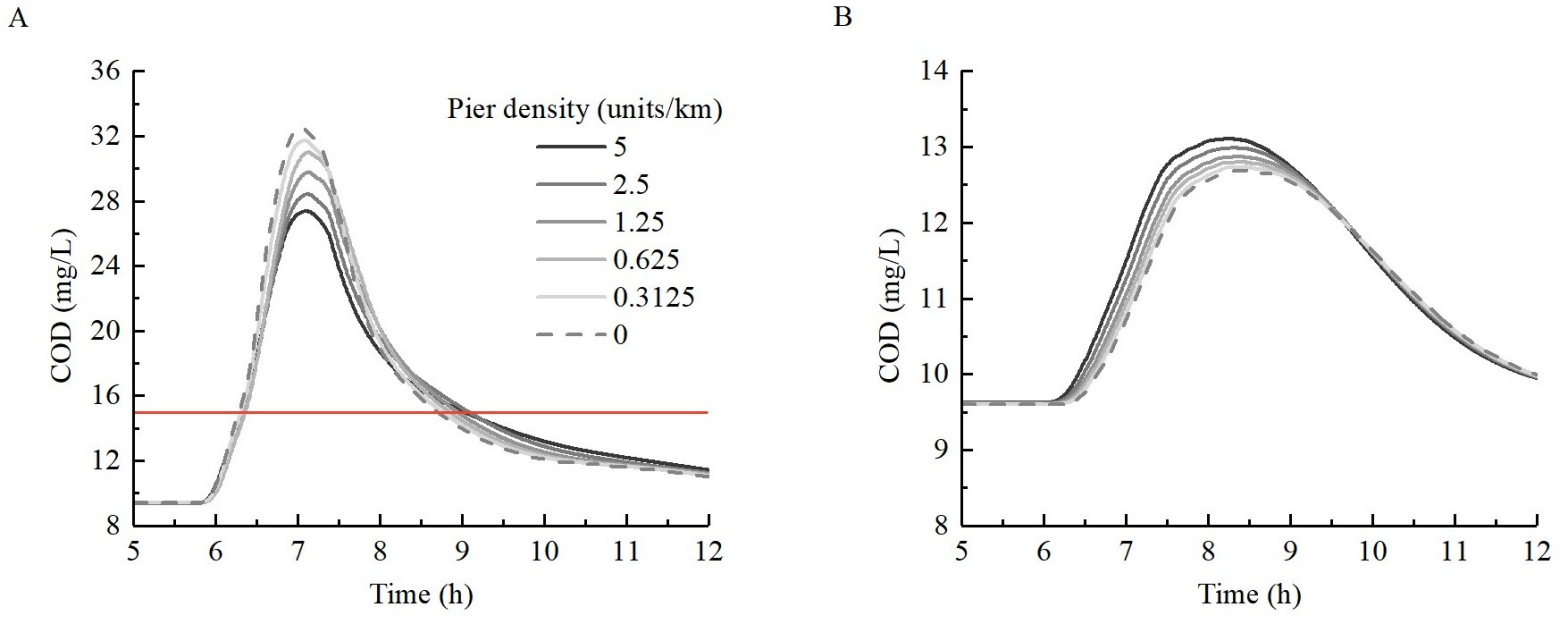
Change in COD concentration at (A) A and (B) B under various pier densities.

The change in COD concentration at locations A and B are shown in Fig 9. It can be observed that the COD concentrations at A and B show asymmetrical profiles with longer concentration-declining phases than concentration-rising phases [29]. Because A is located beside the mainstream area near the right bank, the COD concentration and the variation of which before and after engineering at this location was significantly greater than that of B. The peak COD concentration at A near the right bank decreased with the increase in pier density from 32.5 mg/L when there were no piers (the level IV water quality standard is 30 mg/L) to 27.37 mg/L at the density of 5 units/km. The peak COD concentration of B near the left bank increased slightly with a maximum increase in 1.54 mg/L. From Fig 9, it can also be observed that the retention time of pollutants at A and B extended. However, owing to the large peak concentration, greater changes of the retention time can be seen at A on the right bank. According to the statistics regarding the retention time of pollutants at A exceeding the level II water quality were 2.41 h, 2.44 h, 2.48 h, 2.58 h, 2.73 h, and 2.68 h when the pier densities were 0, 0.31, 0.62, 1.25, 2.5, and 5 units/km, respectively. The maximum increase was 11.2%. The reason for this phenomenon is that the water depth of the shoreline with piers is deep and close to the main channel area. These locations were close to the locations of the main pollutant transfer belt. The transport of pollutants produces a buffer, which inevitably leads to a longer transport duration in the high-concentration area. In summary, in the case of high-concentration pollutant transport in the reach with piers, the waters on the opposite shoreline with piers need to pay attention to both the peak pollutant concentration and increase in the retention time exceeding the water quality level. However, the downstream side on the same bank line should focus on increasing the pollutant retention time.

## Discussion

**Fig 10:**
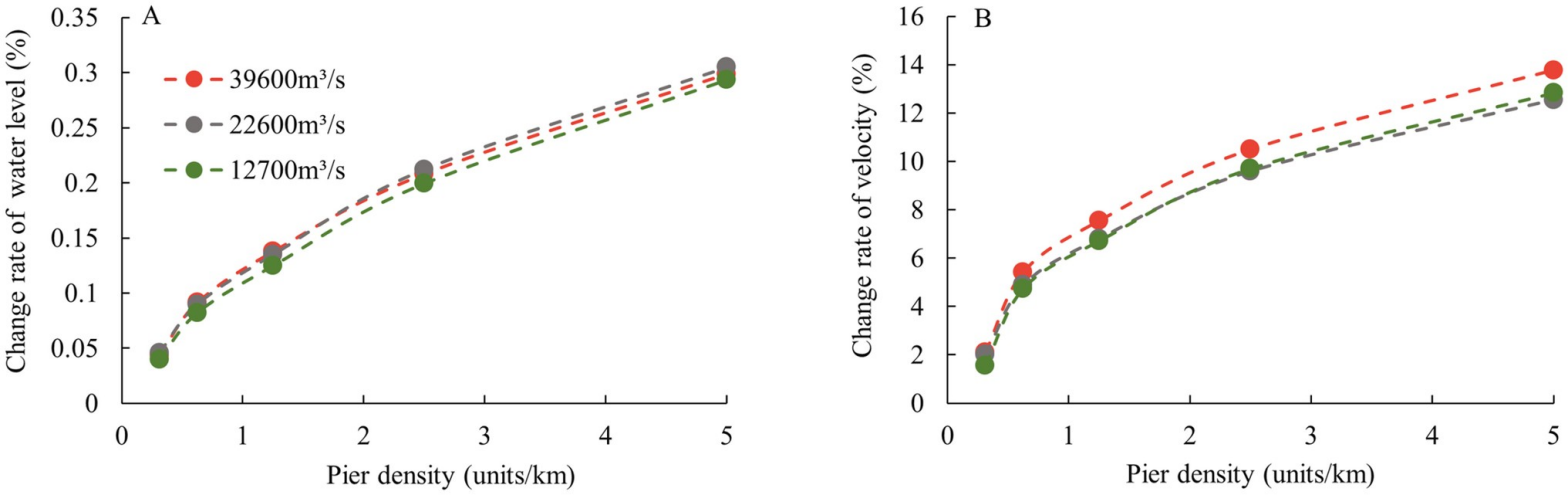
Relationship between the maximum change rates. (A) Water level and (B) velocity and pier density under various discharges.

Fig 10 summarizes the relationship between the maximum magnitude of hydrodynamic change and pier density under various discharges on the grid scale, where the hydrodynamic change refers to the value of the grid with the largest change in the entire model domain. For comparison, the change rate was adopted. The maximum change rate of water level in Fig 10A indicates ($\frac{\text{Maximum}\;\text{water}\;\text{level}\;\text{change}\;\text{with}\;\text{the}\;\text{piers}}{\text{Corresponding}\;\text{location}\;\text{water}\;\text{depth}\;\text{without}\;\text{piers}}$), whereas in Fig 10B, the maximum change rate of velocity in the area where the velocity increases is ($\frac{\text{Maximum}\;\text{velocity}\;\text{change}\;\text{with}\;\text{the}\;\text{piers}}{\text{Corresponding}\;\text{location}\;\text{velocity}\;\text{without}\;\text{piers}}$). It can be observed from the Fig 10 that the relationship between the change rate of water level and pier density under different flow rates was not significantly different, and variations of water level ranged from 0.04% to 0.3%. The relationship between the velocity change rate and pier density is not significantly different under the two levels of medium and dry flows. However, the change rate in flood flow is slightly larger with the value ranging from 1.57% to 13.77%. The analysis of the flow velocity amplitude in the area where the flow velocity decreases shows that the overall change pattern is similar to that observed in Fig 10B, but the amplitude in the decreased velocity area is greater, ranging from −20.48% to −78.92%. Generally, when the pier density is greater than 1.25 to 2.5 units/km, the increase rate in flow conditions will slow down. This is similar to the law that the increase in river resistance gradually slows down with an increase in the density of obstacles in the river [30].

**Fig 11:**
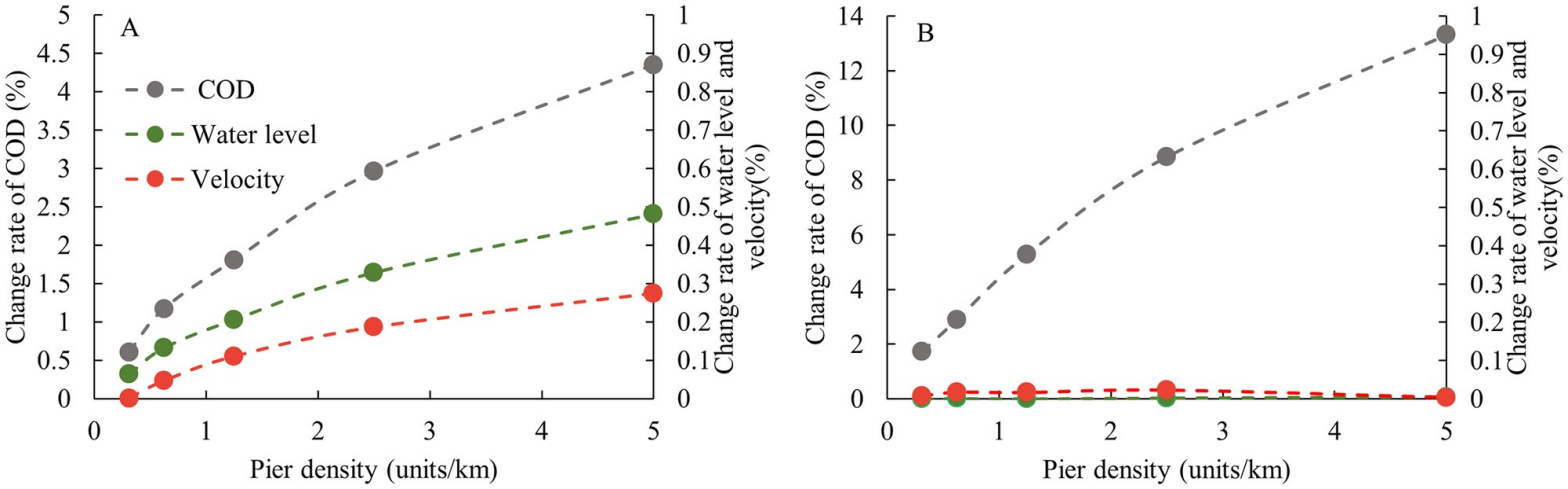
Relationship between the maximum change rates of various parameters. Relationship between the maximum change rate of the average COD concentration, water level, flow velocity, and pier density at the cross-sections of (A) 2# and (B) 5#.

Furthermore, in Fig 11, the cross-sections of 2# and 5# are considered as the study objects and the relationship between the maximum variation of each factor and pier density under the low flow level from the average cross-section scale is summarized. To compare the different factors, the Fig 11 also uses the change rate, the meaning of which is similar to Fig 10, but the average value of cross-section is used. It can be observed that the water level is more sensitive to the response of piers than the velocity at the cross-section of 2# upstream from the engineering area with a maximum change rate of 0.48%. Additionally, the change rate of COD concentration is most sensitive to the increase in the density of piers with a maximum variation of 4.36%. At the cross-section of 5# downstream from the engineering area, the water level is basically unchanged. The average flow velocity of the section has minor changes, but the maximum change of the average COD concentration of the cross-section is 13.31%, which is much larger than that of the 2# cross-section. This phenomenon shows that the impact of piers on the pollutant is greater than that on the flow. Moreover, the affected area is also larger, especially in the downstream cross-section of the project area, where the increase and decrease in flow velocity on the cross-section is almost offset, but the COD concentration is significantly higher than that without piers. Cross-sections of 2# and 5# correspond to the entrance and outlet of the engineering area respectively, and the changes of other cross-sections in the study reach are between sections 2# and 5#. The difference between the two sections shows that the influence of the pier groups on the COD concentration is gradually increasing along the way.

## Conclusions

This study presents the response characteristics of hydrodynamics and dissolved pollutant transport after arranging piers with different densities along the local shoreline using a depth-averaged two-dimensional model.

Although the changes in hydrodynamics are similar under different discharges, the effect of piers on flow regimes varies spatially. The water level upstream of the engineering area increased and decreased at the tail of the project. The flow velocity in the mainstream area at the front of the engineering area increased, whereas the velocity within the engineering area decreased. The range and amplitude of spatial variations of the hydrodynamics increased with the increase in pier density. However, there is a critical value of 1.25 to 2.5 units/km. When the pier density is less than this critical value, this type of cumulative effect is the most significant.

Affected by the spatial variation of hydrodynamics near the engineering area, the variations in pollutant concentration show obvious spatial differences in the transport process downstream from the reach. The peak concentration arrival times upstream and downstream of the engineering area were delayed and advanced, respectively. The pollutant concentration in the mainstream area increased, whereas the pollutant concentration near the shoreline with piers decreased. Under the buffering effect of the pier group on the transport of pollutants, the peak concentration within the project area shows attenuation actions with a longer residence time. Hence, particular attention should be paid to increase the residence time in the region within the downstream of the shoreline with dense piers.

With regard to the change rate, the impact on the shoreline with piers on the transport of pollutants is greater than the impact on hydrodynamic conditions. In addition to flood hazards, during the planning and utilization of the shoreline with piers, the impact of pier density on pollutant transport should also be considered.

## Supporting information

S1 Fig Sketch map showing the hydrological measured section and monitoring points of water quality in calibration stage. (DOCX)

S2 Fig Comparisons between the modeled and observed water levels at typical cross-sections. (DOCX)

S3 Fig Comparisons between the modeled and observed velocities at typical cross-sections under the discharge of 31,800 m^3^/s. (DOCX)

S4 Fig Comparisons between the modeled and observed velocities at typical cross-sections under the discharge of 12,700 m^3^/s. (DOCX)

S5 Fig Comparisons between the modeled and observed COD at different locations under the discharge of 13,627 m^3^/s. (DOCX)

S1 File Water quality data used for the validation of the model. (XLSX)

S2 File Model output of COD with and without piers. (XLSX)
