# Supplementary material for: Simulating the impact of piers on hydrodynamics and pollutant transport: A case study in the Middle Yangtze River
Source: PLoS One. 2021 Dec 1;16(12):e0260527. doi: 10.1371/journal.pone.0260527 (PMC8635386; doi:10.1371/journal.pone.0260527)
Supplement: S1 Fig — (DOCX) [file pone.0260527.s001.docx]

**S1 Fig. Sketch map showing the hydrological measured section and monitoring points of water quality in calibration stage**
