# Supplementary material for: Simulating the impact of piers on hydrodynamics and pollutant transport: A case study in the Middle Yangtze River
Source: PLoS One. 2021 Dec 1;16(12):e0260527. doi: 10.1371/journal.pone.0260527 (PMC8635386; doi:10.1371/journal.pone.0260527)
Supplement: S2 Fig — (DOCX) [file pone.0260527.s002.docx]

**S2 Fig. Comparisons between the modeled and observed water levels at typical cross-sections.** (A) and (B) represent the water level under the discharge of 31,800 m^3^/s, 12,700 m^3^/s, respectively.
