# Supplementary material for: Simulating the impact of piers on hydrodynamics and pollutant transport: A case study in the Middle Yangtze River
Source: PLoS One. 2021 Dec 1;16(12):e0260527. doi: 10.1371/journal.pone.0260527 (PMC8635386; doi:10.1371/journal.pone.0260527)
Supplement: S3 Fig — (DOCX) [file pone.0260527.s003.docx]

**S3 Fig. Comparisons between the modeled and observed velocities at typical cross-sections under the discharge of 31,800 m^3^/s.** (A) 2# cross-section. (B) 4# cross-section. (C) 5# cross-section. (D) 7# cross-section. (E) 8# cross-section. (F) 10# cross-section.
