# Supplementary material for: Simulating the impact of piers on hydrodynamics and pollutant transport: A case study in the Middle Yangtze River
Source: PLoS One. 2021 Dec 1;16(12):e0260527. doi: 10.1371/journal.pone.0260527 (PMC8635386; doi:10.1371/journal.pone.0260527)
Supplement: S4 Fig — (DOCX) [file pone.0260527.s004.docx]

**S4 Fig. Comparisons between the modeled and observed velocities at typical cross-sections under the discharge of 12,700 m^3^/s.** (A) 4# cross-section. (B) 6# cross-section. (C) 7# cross-section. (D) 9# cross-section.
