# Supplementary material for: Simulating the impact of piers on hydrodynamics and pollutant transport: A case study in the Middle Yangtze River
Source: PLoS One. 2021 Dec 1;16(12):e0260527. doi: 10.1371/journal.pone.0260527 (PMC8635386; doi:10.1371/journal.pone.0260527)
Supplement: S5 Fig — (DOCX) [file pone.0260527.s005.docx]

**S5 Fig. Comparisons between the modeled and observed COD at different locations under the discharge of 13,627 m^3^/s.** (A) 20 m from the right bank. (B) 50 m from right bank. (C) 100 m from right bank. (D) 200 m from right bank.
